# A Cross-Sectional Study of Non-communicable Disease Risk Factors Among Non-diabetic and Non-hypertensive Population

**DOI:** 10.7759/cureus.75542

**Published:** 2024-12-11

**Authors:** Rajeshree A Kotawadekar, Vivek B Waghachavare, Alka Gore, Randhir V Dhobale

**Affiliations:** 1 Community Medicine, Bharati Vidyapeeth (Deemed to be University) Medical College and Hospital, Sangli, IND; 2 Biostatistics, Bharati Vidyapeeth (Deemed to be University) Medical College and Hospital, Sangli, IND

**Keywords:** cardiometabolic risk factors, community-based study, epidemiology, high blood pressure, high blood sugar levels, hyperglycemia, noncommunicable diseases, public health hypertension, public health surveillance, screening

## Abstract

Introduction: Noncommunicable diseases, especially diabetes and hypertension, have emerged as significant public health challenges. Regular screening, even among healthy individuals, is essential for early diagnosis and prevention of complications.

Methods: This cross-sectional study was conducted in an urban ward of the Sangli-Miraj-Kupwad municipal corporation in Maharashtra, India, and cluster random sampling was used to collect data. A total of 430 participants aged 10 years and older were enrolled in the study. The institutional committee approved the study, and all guidelines, including consent and confidentiality, were strictly followed throughout the research.

Data collection employed the World Health Organization (WHO) Stepwise approach to surveillance (STEPS) instrument. Descriptive statistics, such as percentage, mean, and standard deviation, were used to summarize the distribution of important variables in the study population. Inferential statistical tests, including unpaired t-tests and analysis of variance (ANOVA), were applied to compare mean blood pressure and blood sugar levels among participants with differing characteristics. Logistic regression was utilized to identify the best predictors for elevated blood pressure and blood sugar levels.

Results: Out of the 430 participants, 366 (85.1%) exhibited risk factors for non-communicable diseases. Elevated blood pressure and blood sugar levels were noted in 220 (51.2%) and 194 (45.1%) subjects, respectively. Age and average systolic blood pressure were the best predictors for random blood sugar levels, while age, lifestyle-related risk factors, religion, overweight, and random blood sugar levels were the best predictors for systolic blood pressure. Marital status and random blood sugar levels were the best predictors for diastolic blood pressure.

Conclusion: Our findings highlight that a substantial proportion of apparently healthy people have elevated blood pressure and blood sugar levels. Hence, regular screening from a young age is recommended.

## Introduction

Globally, non-communicable diseases (NCDs) like hypertension and diabetes mellitus significantly contribute to morbidity and premature mortality. Unhealthy behavioral practices, such as consumption of tobacco, excessive salt, and alcohol, paired with a sedentary lifestyle, can lead to metabolic abnormalities like elevated blood pressure, hyperglycemia, and obesity [1].

After an extensive literature review, Sharma et al. [2] concluded that the NCD risk factors in India require urgent attention. They identified tobacco use, unhealthy diet, sedentary lifestyle, and air pollution as significant contributors to the rising prevalence of NCDs. Additionally, NCDs and their complications negatively impact the lives of patients and their families, posing an important public health challenge. The authors advocated for a multi-pronged approach involving stakeholders and emphasized preventive measures.

Another study from India by Kalyani et al. in Uttarakhand observed that 26% of adult participants had undiagnosed elevated blood pressure [3]. Das et al. from West Bengal found that 21% of the medical students had undiagnosed prehypertension [4]. Similarly, Sahadevan et al. evaluated the National Family Health Survey (NHFS)-5 data and observed that the prevalence rate of undiagnosed diabetes mellitus was 1.22% [5]. In Vietnam, Tho et al. found that 57% of 1841 non-hypertensive and non-diabetic participants had elevated blood sugar levels, and 40% had elevated blood pressure [6]. These findings from multiple studies across diverse geographical areas suggest that a significant proportion of the population has undiagnosed elevated blood pressure and blood sugar levels. If untreated, these persistently elevated levels can result in multiple complications such as loss of vision, ulcers, cardiovascular disorders, renal and liver damage, and stroke [7,8], resulting in increased morbidity and premature mortality.

Overweight and obesity are rising among children and adolescents, affecting over 390 million of them [9]. Leong et al. observed that many overweight or obese adolescents had undiagnosed elevated blood sugar levels and blood pressure [10]. Andes et al. observed that the prevalence of prediabetes among adolescents was 20% [11]. Two multicentric cohort studies from Germany conducted among 15-year-old participants observed elevated blood pressure in 30.7% of the study populations with a high likelihood of missed diagnosis and treatment [12].

To address the challenge posed by hypertension and diabetes, it is crucial to target at-risk healthy individuals proactively. Identifying and managing prediabetic and prehypertensive individuals can significantly delay the onset of these diseases and their complications. This strategy involves vigilant monitoring of adolescents for elevated blood pressure and blood sugar levels, and introducing protective measures at an early stage. Azegami et al. [13] noted that many factors negatively affecting a child's health are precursors of adult hypertension. They emphasized the challenges in early identification and prevention of the risk factors for elevated blood pressure.

Research on undiagnosed prediabetes and prehypertension among apparently-healthy Indian populations, especially children and youth, is limited. Our study aimed to observe these conditions among seemingly-healthy individuals from the Sangli-Miraj-Kupwad municipal corporation in Maharashtra, India. We hope to evoke the interest of researchers to conduct similar studies.

We presented this study as a poster at the Epidemiology Foundation of India's Annual National Conference, EFICON 2023, on September 30, 2023.

## Materials and methods

We conducted this cross-sectional study in a randomly selected urban ward of the Sangli-Miraj-Kupwad municipal corporation, Maharashtra, India, from March 2021 to August 2022. Based on the 33.1% prevalence observed by Sarma et al. (2019) in Kerala [14], we calculated a sample size of 340 [n=z2pq/d2 with z=1.96, p=0.331, q=0.669; confidence level of 95%; and the desired margin of error (d) of 5%]. We then employed cluster random sampling, resulting in 430 study participants.

The institutional ethics committee of Bharati Vidyapeeth (Deemed to be University) Medical College and Hospital, Sangli approved the study [BV(DU)MC&H)/Sangli/IEC/Dissertation2020-21/357]. The study participants were adolescents (10-19 years) and adults who were not known cases of diabetes or hypertension. Residents of the area who were present during data collection were invited to participate. Critically ill and bedridden patients and individuals with intellectual disabilities were excluded from the study. Informed consent was obtained from the adults, and parental consent and individual assent were acquired from adolescents.

The participants were briefed on the research, its significance, voluntary participation, and confidentiality. Data was collected using a pre-tested and pre-validated interview schedule based on the World Health Organization STEPwise approach to surveillance (WHO STEPS) instrument [15]. Data collection involved interviews, clinical and anthropometric examinations, blood pressure assessments, and random blood sugar level investigations. The interview schedule had basic socio-demographic information of the participants (e.g., age, gender, urban-rural residence, occupation, dietary preferences, socio-economic status using the modified BG Prasad classification scale [16], etc.) and information on the presence of modifiable risk factors for noncommunicable diseases (e.g., tobacco consumption, alcohol use, physical activity, stress, etc.). Height and weight were measured to calculate body mass index (BMI) and identify overweight individuals. A glucometer was used to measure the participants' random blood glucose levels. The procedure involved the following steps: All aseptic precautions were followed, and a small drop of blood was obtained from a finger using a new lancet for every participant. The blood drop was applied to a test strip inserted into the glucometer, and the reading was recorded. Blood pressure was measured three times (immediately, the next morning, and a day after in the morning) in the right arm of the seated participant after a minimum of five minutes rest. Participants with elevated blood pressure (reference WHO criteria for age) were allowed to rest for 30 minutes, after which the blood pressure was measured again. All the participants were counseled on risk factors, complications, and the prevention of diabetes and hypertension. Those with elevated blood pressure or blood sugar levels were referred to clinicians.

We employed Microsoft 365 (Microsoft Corp., Redmond, WA, US) and IBM SPSS Statistics for Windows, Version 22 (Released 2013; IBM Corp., Armonk, New York, United States) for data analysis. In descriptive statistics, we applied frequencies with percentages to qualitative data, while for quantitative data, we used mean with standard deviation (SD). As all the variables were normally distributed (verified using the Shapiro-Wilk test, P>0.05), we used unpaired t-tests and ANOVA (analysis of variance) to compare means between groups. Binary logistic regression identified predictors of elevated random blood sugar levels and systolic or diastolic blood pressure.

## Results

Of the 430 participants, 148 (34.4%) were males, and 282 (65.6%) were females. The mean age of participants was 39.15 years (SD±18.2 years) with a range of 10 to 87 years. Considering the age group, 84 (19.5%) participants were adolescents (10-19 yrs), 282 (65.6%) were adults (20-59 yrs), and 64 (14.9%) were elderly (≥60 yrs). Most of the participants, i.e., 357 (83%), belonged to the Hindu religion, while 73 (17%) belonged to the Muslim faith. Considering their marital status, 284 (66%) of them were married, 116 (27%) were single and 30 (7%) were widowed. The education status of the study participants was as follows: 21 (4.9%) were illiterate, 91 (21.2%) had completed primary school, 292 (67.9%) had finished secondary school, and 26 (6%) had attended college. Most participants, 236 (54.9%), belonged to socioeconomic Class II. This was followed by 114 (26.5%) belonging to Class I, 78 (18%) to Class III, and two (0.5%) to Class IV.

Lifestyle-related risk factors were reported as follows (factor, number of participants, percentage): smoking 23 (5.3%), tobacco chewing 43 (10%), alcohol consumption 2 (0.5%), extra salt in diet 104 (24.2%), sedentary lifestyle 85 (19.8%), and overweight 139 (32.3%). Considering the number of modifiable risk factors, 64 (14.9%) participants reported no risk factor, while 366 (85.1%) reported one or more risk factors.

On examination, 194 (45.1%) had elevated random blood sugar levels, and 220 (51.2%) had elevated blood pressure. The mean random blood sugar level was 137.88 (SD±25.186) mg/dl. The mean systolic and diastolic blood pressure were 128.56 (SD±23.689) mmHg and 76.77 (SD±7.203) mmHg, respectively. Table 1 presents the distribution of these parameters in the study participants. Among the adolescent participants, a random elevated blood sugar level was observed in one (1.2%) participant, elevated systolic blood pressure in one (1.2%), and elevated diastolic blood pressure in four (4.8%) participants.

**Table 1:** Frequency distribution table for random blood sugar level, and systolic and diastolic blood pressure

Clinical variables	Number of participants	Percentage (%)
Random blood sugar level		
≤140 mg/dl	236	54.9
141-199 mg/dl	189	44
≥200 mg/dl	5	1.2
Systolic blood pressure		
≤ 120 mmHg	232	54
121-140 mmHg	84	19.5
>140 mmHg	114	26.5
Diastolic blood pressure		
≤80 mmHg	313	72.8
81-90 mmHg	92	21.4
>90 mmHg	25	5.8

We compared mean random blood sugar levels, and systolic and diastolic blood pressure across various sociodemographic, lifestyle-related, and clinical variables using an unpaired t-test and ANOVA. Mean random blood sugar level was significantly different across multiple variables (Table 2) and was significantly higher in the participants with elevated blood pressure. On the other hand, there was no significant difference in mean random blood sugar level across certain parameters like gender, socioeconomic status, smoking status, alcohol consumption, and sedentary lifestyle. Similarly, the mean systolic blood pressure was significantly different across some of the parameters assessed (Table 3). In contrast, there was no significant difference in systolic blood pressure based on gender, smoking status, alcohol consumption, extra salt in the diet, sedentary lifestyle, and type of diet. Lastly, mean diastolic blood pressure significantly differed across several parameters as shown in Table 4. There was no significant difference in diastolic blood pressure in the case of variables such as gender, religion, smoking status, alcohol consumption, and type of diet.

**Table 2:** Comparison of mean random blood sugar levels across various variables *One-way ANOVA used; ANOVA, Analysis of variance; SD, Standard deviation; t, Unpaired t-test value; F, F-statistic on ANOVA

Sociodemographic variables		Mean random blood sugar level (±SD)	t/F	p-value
Religion	Hindu	135.93 (±23.751)	12.951	0.000
	Muslim	147.41 (±29.656)		
Age group	Adolescents	112.62 (±12.182)	108.52*	0.000
	Adults	139.95 (±21.506)		
	Elderly	161.89 (±24.604)		
Marital status	Married	142.94 (±22.084)	116.391*	0.000
	Single	116.53 (±14.839)		
	Widow	172.47 (±20.989)		
Education	Illiterate	170.1 (±23.841)	23.478*	0.000
	Primary school	147.76 (±22.882)		
	Secondary school	133.1 (±24.004)		
	College	130.88 (±17.154)		
Chewing tobacco	No	135.97 (±24.949)	23.3	0.000
	Yes	155.02 (±20.594)		
Extra salt in the diet	No	136.15 (±25.528)	6.41	0.012
	Yes	143.29 (±23.384)		
Overweight	No	131.2 (±22.577)	74.14	0.000
	Yes	151.86 (±24.691)		
Lifestyle-related risk factors	No	128.46 (±21.704)	38.35	0.000
	Yes	143.4 (±25.483)		
Type of diet	Mixed	138.85 (±25.125)	12.23	0.001
	Veg	119.82 (±19.075)		
Blood pressure	Not elevated	122.67 (±18.181)	229.17	0.000
	Elevated	152.39 (±22.222)		

**Table 3:** Comparison of mean systolic blood pressure across various variables *One-way ANOVA used; ANOVA, Analysis of variance; SD, Standard deviation; t, Unpaired t-test value; F, F-statistic on ANOVA

Sociodemographic variables		Mean systolic blood pressure (±SD)	t/F	p-value
Religion	Hindu	126.75 (±18.154)	12.539	0.000
	Muslim	137.38 (±40.236)		
Age group	Adolescents	110.49 (±6.910)	61.183*	0.000
	Adults	129.36 (±24.480)		
	Elderly	148.72 (±15.310)		
Marital status	Married	132.12 (±24.717)	62.121*	0.000
	Single	112.84 (±7.764)		
	Widow	155.63 (±15.158)		
Socio-economic status	Class I	125.12 (±15.64)	4.219*	0.005
	Class II	127.69 (±19.22)		
	Class III	136.56 (±39.35)		
	Class IV	114 (±8.49)		
Education	Illiterate	150.76 (±19.565)	13.51*	0.000
	Primary school	136.42 (±16.75)		
	Secondary school	125.23 (±25.253)		
	College	120.46 (±7.35)		
Chewing tobacco	No	127.06 (±23.987)	15.92	0.000
	Yes	142 (±15.441)		
Overweight	No	124.76 (±25.257)	24.43	0.000
	Yes	136.51 (±17.601)		
Lifestyle-related risk factors	No	121.81 (±16.682)	21.47	0.000
	Yes	132.52 (±26.196)		

**Table 4:** Comparison of mean diastolic blood pressure across various variables *One-way ANOVA used; ANOVA, Analysis of variance; SD, Standard deviation; t, Unpaired t-test value; F, F-statistic on ANOVA

Sociodemographic variables		Mean diastolic blood pressure (±SD)	t/F	p-value
Age group	Adolescents	74.06 (±4.855)	7.645*	0.001
	Adults	77.41 (±6.687)		
	Elderly	77.44 (±7.713)		
Marital status	Married	77.77 (±7.673)	11.155*	0.000
	Single	74.14 (±5.204)		
	Widow	77.57 (±6.897)		
Socio-economic status	Class I	74.41 (±6.91)	6.545*	0.000
	Class II	77.28 (±7.25)		
	Class III	78.65 (±6.73)		
	Class IV	79 (±5.66)		
Education	Illiterate	75.24 (±4.898)	4.148*	0.006
	Primary school	79.05 (±7.549)		
	Secondary school	76.16 (±7.181)		
	College	76.88 (±6.364)		
Chewing tobacco	No	76.34 (±6.89)	14.45	0.000
	Yes	80.67 (±8.739)		
Extra salt in the diet	No	76.21 (±7.059)	8.46	0.004
	Yes	78.55 (±7.394)		
Overweight	No	75.67 (±6.833)	22.35	0.000
	Yes	79.09 (±7.428)		
Lifestyle-related risk factors	No	75.3 (±6.643)	10.78	0.001
	Yes	77.64 (±7.389)		

Though not statistically significant, males had higher mean random blood sugar levels, and systolic and diastolic blood pressure than females. All three parameters were lowest in adolescents, followed by adults and then the elderly. The Muslim participants had higher mean random blood sugar levels and systolic blood pressure compared to the Hindu participants. The mean of these three parameters was highest among widowed participants, followed by married and single participants. Considering socioeconomic class, higher mean random blood sugar levels and systolic blood pressure were observed in class III, while mean diastolic blood pressure was highest in class IV. Illiterate participants had higher mean random blood sugar levels and systolic blood pressure, and these parameters had a reducing trend with increasing education. The mean of all the three parameters was higher among smokers, and those who chew tobacco, consume alcohol or extra salt in their diet, those having mixed diets, and in overweight participants.

As presented in Tables 5-7, binary logistic regression using the Wald method was applied to the dependent factors: random blood sugar levels, and systolic and diastolic blood pressure. Age and average systolic blood pressure were the best predictors for random blood sugar levels, while age, lifestyle-related risk factors, religion, overweight and random blood sugar levels were the best predictors for systolic blood pressure. Marital status and random blood sugar levels were the best predictors for diastolic blood pressure.

**Table 5:** Binary logistic regression models for random blood sugar levels Model accuracy 71.9; B, Regression coefficient; Wald, Wald’s coefficient; CI, Confidence interval. For all the variables, the degree of freedom df=1.

Variables	B	Standard Error	Wald	p-value	Odds ratio (95% CI)
Age	0.05	0.011	21.842	0.000	1.051 (1.029-1.073)
Marital status	-0.38	0.264	2.07	0.15	0.684 (0.408-1.148)
Lifestyle-related risk factors	0.205	0.337	0.369	0.543	1.228 (0.634-2.378)
Religion	0.339	0.343	0.975	0.323	1.403 (0.716-2.747)
Type of diet	-0.871	0.834	1.09	0.297	0.419 (0.082-2.148)
Overweight	0.057	0.341	0.028	0.867	1.059 (0.543-2.064)
Average systolic BP	0.065	0.012	27.888	0.000	1.067 (1.042-1.093)
Average diastolic BP	0.013	0.02	0.444	0.505	1.013 (0.975-1.053)
Constant	-10.62	2.023	27.56	0	0

**Table 6:** Binary logistic regression models for systolic blood pressure Model Accuracy 80.5; B, Regression coefficient; Wald, Wald’s coefficient; CI, Confidence interval. For all the variables, the degree of freedom df=1.

Variables	B	Standard Error	Wald	p-value	Odds ratio (95% CI)
Age	0.091	0.012	53.477	0.000	1.096 (1.069-1.123)
Marital Status	-0.236	0.32	0.545	0.461	0.79 (0.421-1.479)
Lifestyle-related risk factors	1.042	0.375	7.74	0.005	2.835 (1.361-5.908)
Religion	-0.818	0.39	4.407	0.036	0.441 (0.206-0.947)
Type of Diet	-0.577	0.887	0.423	0.515	0.561 (0.099-3.195)
Overweight	1.092	0.382	8.151	0.004	2.98 (1.408-6.306)
Random BSL	0.045	0.008	30.869	0.000	1.046 (1.03-1.063)
Constant	-9.34	1.648	32.114	0	0

**Table 7:** Binary logistic regression models for diastolic blood pressure Model Accuracy 86; B, Regression coefficient; Wald, Wald’s coefficient; CI, Confidence interval. For all the variables, the degree of freedom df=1.

Variables	B	Standard Error	Wald	p-value	Odds ratio (95% CI)
Age	0.015	0.009	2.853	0.091	1.015 (0.998-1.032)
Marital status	-0.478	0.195	5.996	0.014	0.62 (0.423-0.909)
Lifestyle-related risk factors	0.603	0.31	3.767	0.052	1.827 (0.994-3.357)
Religion	-0.599	0.33	3.293	0.07	0.549 (0.288-1.049)
Type of diet	-0.651	0.785	0.688	0.407	0.522 (0.112-2.428)
Overweight	0.252	0.284	0.788	0.375	1.287 (0.737-2.246)
Random blood sugar level	0.018	0.006	8.071	0.004	1.018 (1.006-1.031)
Constant	-2.605	1.178	4.886	0.027	0.074

## Discussion

In a study of the tribal population from Madhya Pradesh, Chamka et al. (2017) found that 26% had high blood pressure [17]. The proportion of elevated blood pressure was much higher in our study. As discussed by Chamka et al., this difference can be attributed to the observation that most tribals are involved in heavy manual labor and the proportion of obesity is lower among them. However, similar to our findings, Chamka et al. observed a higher prevalence among males, an increasing trend of higher blood pressure with age, and significantly higher blood pressure among participants who were illiterate, chewed tobacco, were overweight, and consumed higher salt.

Kapil et al. (2018) researched the geriatric population from the hilly areas of Uttarakhand, India [18]. In their study, the mean age of the male and female participants was 69.5 (±7.4) and 67.8 (±7.2) years, respectively. Elevated systolic blood pressure was observed in 82.7% of the participants, diastolic blood pressure in 65.5%, and fasting glucose level in 25%. Compared to their study, we had younger participants at a mean age of 39.15 (±18.2) years. Hence, elevated blood pressure was observed in fewer participants in our study. In the case of blood sugar levels, they conducted a fasting blood glucose test, which is more specific than the random glucose test. Thus, it is difficult to compare the results.

Sarma et al. (2019) observed that the prevalence of elevated blood pressure and fasting glucose among urban residents in Kerala, India was 33.1% and 19.8%, respectively [14]. These values were lower than our findings. The study by Sarma et al. was an extensive multicentric survey. In contrast, our study was restricted to one area from a selected city and we accept this limitation. The age range of the participants in the study by Sarma et al. was 18-69 years compared to our study (10-87 years), which may also contribute to the differences in observations. On the other hand, in both studies, over 80% of the participants exhibited one or more risk factors for NCDs.

Rajbhabdari et al. (2020) studied 3882 participants from 32 sites in Bangalore, India [19]. They observed elevated random blood sugar levels in one-third of the participants, which was lower than our findings. However, like our study, they also observed lower blood sugar levels and systolic blood pressure among vegetarians.

Kurjogi et al. (2021) studied patients >18 years old visiting NCD OPDs in Hubballi and Dharwad [20]. They observed that 65.6% of the patients had elevated blood pressure. This proportion was higher than our observation, as their study was conducted among OPD patients while we collected our data from the community. Like our observation, Kurjogi et al. too concluded that males and those who chewed tobacco had a higher prevalence of hypertension.

Mathur et al. (2022) [21] studied the data from the National Noncommunicable Disease Monitoring Survey (NNMS) (2017-2018). They observed that out of the 2879 urban participants with no previous history of diabetes, 1088 (37.8%) had elevated fasting blood sugar levels. This prevalence was slightly lower than that seen in our study. This could be attributed to the multicentric nature of the NNMS and the use of a more specific screening test i.e., fasting blood sugar level. Like our study, Mathur et al. observed the association between impaired blood glucose levels with age group, tobacco use, BMI levels, and raised blood pressure.

In a study from Trichy conducted among 878 school-going children aged 6 to 13, increased blood pressure was observed in 5.58% of participants [22]. Similarly, we observed elevated blood pressure in 4.8% of adolescents from the urban community. So, both studies highlighted that a significant proportion of children and adolescents have elevated blood pressure.

We acknowledge some limitations of the study. A multicentric study would provide much better understanding of the problem. Due to economic constraints, we could not conduct other biochemical blood investigations, such as lipid profile, HbA1c, etc., which would have been more suited for understanding the risk factors.

## Conclusions

A large proportion of our participants were obese or overweight, with elevated random blood sugar levels and blood pressure. These elevations were also more common among the illiterate, widowed, and middle-class participants in our study. Many people are unaware of their elevated blood pressure or blood sugar levels. Screening for these risk factors should start early, preferably in adolescence or even younger age groups.

Management of at-risk individuals should focus on lifestyle modifications such as proper diet, cessation of smoking, regular exercise, adequate weight control, and reduction in alcohol consumption. Similarly, community research across all age groups and research in primordial and primary prevention of NCDs should be encouraged.
